# A missed opportunity – consequences of unknown levetiracepam pharmacokinetics in a peritoneal dialysis patient

**DOI:** 10.1186/1471-2369-15-49

**Published:** 2014-04-16

**Authors:** Svenja K Bahte, Marcus Hiss, Ralf Lichtinghagen, Jan T Kielstein

**Affiliations:** 1 Department of Nephrology and Hypertension and Institute for Clinical Chemistry, Medical School Hannover, Carl-Neuberg-Strasse 1, Hannover 30625, Germany

## Abstract

**Background:**

Levetiracetam is a frequently used drug in the therapy of partial onset, myoclonic and generalized tonic-clonic seizures. The main route of elimination is via the kidneys, which eliminate 66% of the unchanged drug as well as 24% as inactive metabolite that stems from enzymatic hydrolysis. Therefore dose adjustments are needed in patients with chronic kidney disease stage 5 D, i.e. patients undergoing dialysis treatment. In this patient population a dose reduction by 50% is recommended, so that patients receive 250–750 mg every 12 hours. However “dialysis” can be performed in using different modalities and treatment intensities. For most of the drugs pharmacokinetic data and dosing recommendations for patients undergoing peritoneal dialysis are not available. This is the first report on levetiracetam pharmacokinetics in a peritoneal dialysis patient.

**Case presentation:**

A 73-y-old Caucasian male (height: 160 cm, weight 93 kg, BMI 36.3 kg/m^2^) was admitted with a Glasgow Coma Scale of 10. Due to diabetic and hypertensive nephropathy he was undergoing peritoneal dialysis for two years. Eight weeks prior he was put on levetiracetam 500 mg twice daily for suspected partial seizures with secondary generalization. According to the patient’s wife, levetiracetam lead to fatigue and somnolence leading to trauma with fracture of the metatarsal bone. Indeed, even 24 hours after discontinuation of levetiracetam blood level was still 29.8 mg/l (therapeutic range: 12 – 46 mg/l). Fatigue and stupor had disappeared five days after discontinuation of the levetiracepam. A single dose pharamockinetic after re-exposure showed an increased half life of 18.4 hours (normal half life 7 hours) and levetiracetam content in the peritoneal dialysate. Both half-life and dialysate content might help to guide dosing in this patient population.

**Conclusion:**

If levetiracetam is used in peritoneal dialysis patients it should be regularly monitored to avoid supratherapeutic levels that could lead to severe sequelae.

## Background

Several guidelines list levetiracetam as the drug of first choice for adult patients with partial seizures with or without secondary generalization and as second choice for patients in the epileptic status. Levetiracetam (molecular weight 170.2 Da) is rapidly absorbed after oral ingestion with bioavailability of almost 100%. Peak concentrations are reached 1.3 hours after ingestion. Neither levetiracetam nor its primary metabolites are bound to plasma proteins. Its volume of distribution is 0.5 – 0.7 l/kg. The elimination half-life in adults is 7 ± 1 hours. Only about a quarter of the dose is metabolised by enzymatic hydrolysis in the blood. The cumulated renal excretion rate of levetiracetam is 66% in the first 48 hours [1]. These pharmacokinetic coordinates vividly illustrate that meticulous attention has to be paid if patients with chronic kidney disease are treated [2]. It has been advocated to decrease the dose in parallel with the decline of the glomerular filtration rate [1]. For patients “on dialysis” the package insert suggests a dose of 500–1000 mg once a day. As the small unbound molecule is easily removed by “dialysis” a supplemental dose of 250–500 mg after dialysis is suggested. Despite the fact that levetiracetam was approved 13 years ago, there are no data for dosing in peritoneal dialysis patients. We therefore analysed pharmacokinetic data of levetiracetam in a patient on peritoneal dialysis for treatment of partial seizures.

## Case presentation

A 73-y-old Caucasian male was admitted to our tertiary care hospital to undergo elective angioplasty due to peripheral artery disease Fontaine’s stage IV. Diabetic and hypertensive nephropathy led to CK5D, i.e. chronic dialysis. He had been undergoing peritoneal dialysis treatment for two years. His past medical history included Insulin-dependent Diabetes mellitus type II and heart failure New York Heart Association stage III due to severe ischemic cardiomyopathy. He was status post aortocoronary bypass operation and had a cardioverter-defibrillator due to recurrent monomorphic ventricular tachycardia.

On admission the patient complained about fatigue and stupor. His wife reported that that his agitated and at times hostile temper had recently completely subsided. Relieved at first, the wife of the patient was than worried as this hallmark of his character had remained constant since they met more than 50 years ago. A thorough history revealed this new calm and at times sleepy state coincided with the start of levetiracetam treatment. The patient received a dose of 500 mg bid due to suspected partial seizures with secondary generalization eight weeks to the recent admission. A neurological workup at that time showed however no pathological findings. Due to the severe fatigue our patient stumbled and fractured his metatarsal bone of his left digitus V a week prior to admission.

**Figure 1:**
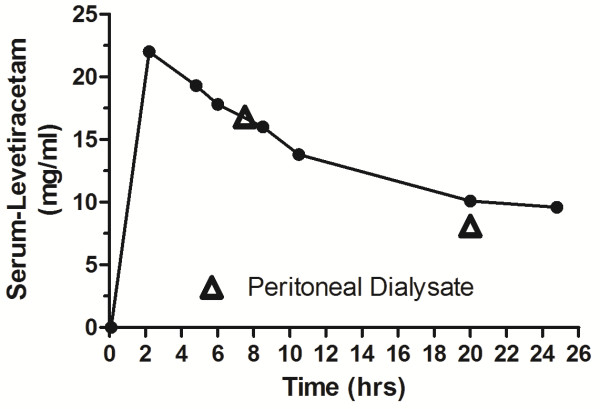
**Levetiracetam pharmacokinetics after a single oral dose of 500 mg.** The two concentrations in the peritoneal dialysate are depicted as triangles.

On admission physical examination showed an obese (height: 160 cm, weight 93 kg, BMI 36.3 kg/m^2^) patient with a Glasgow Coma Scale of 10 and with ulcerations of both legs. Neither popliteal, tibial nor peroneal pulses were palpable. His blood pressure was 135/75 mmHg, heart rate 65 bpm. He presented with anemia (hemoglobin of 8.7 [13.5-17.5] g/dl) and an elevated c-reactive protein of 46 [<8] mg/l. Percutaneous transluminal angioplasty could be performed with good result and without complication. As fatigue and drowsiness did not improve over time, we assumed an overdosing of the antiepileptic drug levetiracetam with typical symptoms of overdose, particularly as vital signs, especially blood pressure, were normal and evidence of cardiac instability were missing. Twentyfour hours after discontinuation of levetiracetam we found levetiracetam-levels still at about 29.8 mg/l (therapeutic range: 12 – 46 mg/l). Five days after discontinuation the level was at 2 mg/l. Unfortunately there were neither blood samples at the time of trauma, nor between discontinuation of the drug and day 5 after discontinuation available, which would have allowed a stronger conclusion in terms of causality. Clinically, however fatigue and stupor had disappeared five days after discontinuation of the levetiracepam. In order to establish the assumed accumulation/overdose of levetiracetam we re-exposed the patient to the drug. After discontinuing levetiracetam for 7 days, a single dose of 500 mg levetiracetam was administered after end of automated peritoneal dialysis (APD). Blood was taken before ingestion and at regular intervals until 24 hours after ingestion as well as peritoneal fluid before ingestion and 7.5 h and 20 h after ingestion in order to study pharmacokinetics in peritoneal dialysis (Figure 1). Samples were centrifuged at 2800xg for 5 min at 4°C and stored at −80°C until analysis. Levetiracetam levels were measured using an HPLC method. In brief, after a liquid-liquid extraction of 300 μl serum to eliminate lipophile components the samples were applied to a reversed phase HPLC with UV detection. The detection limit of levetiracetam was 1 mg/l and the linearity of the method was from 0–100 mg/l. Intra-assay CV was 3.1% (pool serum with 15 mg/l; n = 10), Inter-assay precision (n = 8) was 7.2% (15 mg/l), and 6% (30 mg/l), respectively. Levetiracetam half life after a single oral dose of 500 mg was found to be 18.4 hours, which is more than doubled as compared to patients with normal renal function (half life 7 hours). Serum levels and peritoneal fluid levels were nearly equivalent at two different time period.

## Conclusions

Drug dosing in patients with chronic kidney disease can be a challenging task, especially for those drugs in which neither a physiological readout (blood pressure) nor laboratory data (blood glucose) are available. Peritoneal dialysis is a rather infrequently used dialysis modality. According to the 2012 USRDS report (http://www.usrds.org) less than 30,000 patients in the US undergo peritoneal dialysis as compared to 400,000 patients on hemodialysis, which is below the global rate of 12% [3]. Choice of dialysis modality is however very heterogenous and influenced by multiple factors which explains extremes such as a peritoneal dialysis rate of 0% in Luxemburg and a rate of 49% in New Zealand among patients on maintenance dialysis [3]. In general, the quantity of drugs removed during peritoneal dialysis is substantially lower than that during hemodialysis, and thus, the supplemental administration of drugs, even when they are efficiently removed during hemodialysis, is not necessary in patients receiving continuous ambulatory peritoneal dialysis (CAPD) [4]. There are only limited data on how newer antiepileptic drugs are handled by the kidney and especially how the different modes and intensities affect those drugs [5]. In a situation like this even a single case like ours can enhance the quality of pharmacokinetic information available to clinicians, as recently asked for by a KDIGO working group [6]. Antiepileptic drugs are frequently used in patients with renal impairment. Safe and effective treatment requires attention to changes in pharmacokinetics and knowledge about the effect of extracorporeal treatment methods in terms of elimination of these drugs. Therapeutic drug monitoring can be a valuable aid [7]. For levetiracetam most individuals display optimal response to treatment with trough serum levels 12 to 46 mg/l. Some individuals may respond well outside of this range, or may display toxicity within the therapeutic range, thus interpretation should include clinical evaluation [8]. Toxic levels have not been well established but anecdotal reports show that overdose is facilitated if chronic kidney disease is present [9]. For patients in CKD 5 D, i.e. patient undergoing dialysis PubMed (http://www.ncbi.nlm.nih.gov/pubmed) does not list a single pharmacokinetic study. The package insert such as reference books and online resources recommend a dose of 250–500 mg bid and an additional dose of 250–500 mg after “dialysis”.

Our data from a peritoneal dialysis patient suggest that a dose as low as 500 mg bid has the potential of causing severe side effects. As dosing guidelines for patients on thrice weekly hemodialysis should not be simply transferred to patients undergoing daily peritoneal dialysis it is also not understandable how some online-resources advice to reduce the dose of levetiracetam in pediatric peritoneal dialysis patients by 50%. Our case illustrates that therapeutic drug monitoring should be used in this patient population whenever possible. This opportunity was missed in the reported case and could probably have avoided the severe side effects of the levetiracepam overdose.

## Consent

Written consent was obtained from the patient for publication of this study. Ethical approval was for reporting this case was obtained from the Medical School Hannover.

## Competing interests

The authors declare that they have no competing interests.

## Authors’ contributions

RL conducted the measurement of levetiracetam. SKB, MH, and JTK were the treating physicians of the patient reported. RL and JTK evaluated the test results. All of the authors have participated in the discussion and in writing of the submitted manuscript. All authors read and approved the final manuscript.

## Pre-publication history

The pre-publication history for this paper can be accessed here:

http://www.biomedcentral.com/1471-2369/15/49/prepub
